# Deep Segmentation Networks for Segmenting Kidneys and Detecting Kidney Stones in Unenhanced Abdominal CT Images

**DOI:** 10.3390/diagnostics12081788

**Published:** 2022-07-23

**Authors:** Dan Li, Chuda Xiao, Yang Liu, Zhuo Chen, Haseeb Hassan, Liyilei Su, Jun Liu, Haoyu Li, Weiguo Xie, Wen Zhong, Bingding Huang

**Affiliations:** 1 College of Big Data and Internet, Shenzhen Technology University, Shenzhen 518118, China; 1910412006@email.szu.edu.cn (D.L.); 2070416011@stumail.sztu.edu.cn (C.X.); haseeb@sztu.edu.cn (H.H.); suliyilei@sztu.edu.cn (L.S.); 2100411010@stumail.sztu.edu.cn (H.L.); 2 Wuerzburg Dynamics Inc., Shenzhen 518118, China; zhuo.chen@wuerzburg-dynamics.com (Z.C.); jun.liu@wuerzburg-dynamics.com (J.L.); weiguo.xie@wuerzburg-dynamics.com (W.X.); 3 Department of Urology, The First Affiliated Hospital of Guangzhou Medical University, Guangzhou 510120, China; lyurology@gmail.com

**Keywords:** semantic segmentation networks, kidney segmentation, kidney detection, kidney stone segmentation, kidney stone detection, computed tomography

## Abstract

Recent breakthroughs of deep learning algorithms in medical imaging, automated detection, and segmentation techniques for renal (kidney) in abdominal computed tomography (CT) images have been limited. Radiomics and machine learning analyses of renal diseases rely on the automatic segmentation of kidneys in CT images. Inspired by this, our primary aim is to utilize deep semantic segmentation learning models with a proposed training scheme to achieve precise and accurate segmentation outcomes. Moreover, this work aims to provide the community with an open-source, unenhanced abdominal CT dataset for training and testing the deep learning segmentation networks to segment kidneys and detect kidney stones. Five variations of deep segmentation networks are trained and tested both dependently (based on the proposed training scheme) and independently. Upon comparison, the models trained with the proposed training scheme enable the highly accurate 2D and 3D segmentation of kidneys and kidney stones. We believe this work is a fundamental step toward AI-driven diagnostic strategies, which can be an essential component of personalized patient care and improved decision-making in treating kidney diseases.

## 1. Introduction

Chronic kidney disease (CKD) causes a gradual loss of kidney functions and affects 26 million patients in the US [1]. Nephrolithiasis (the process of forming kidney stones-renal calculi) is also a highly prevalent kidney disorder affecting approximately one in eleven people [2]. Kidney or urinary stones occur when urine minerals separate and crystallize due to a chemical imbalance [3]. The prevalence of nephrolithiasis is increasing [4], and imaging modalities such as unenhanced computed tomography (CT), enhanced CT, magnetic resonance imaging (MRI), and ultrasound are essential for accurate and timely diagnosis. Recently, artificial intelligence (AI) and computer vision (CV) algorithms have been applied to these imaging modalities [5,6], which play a promising role in triaging cases [7] and improving workflow in emergencies [8]. These algorithms are essential for renal disease detection and monitoring, giving insight into renal function and various renal pathologies [9]. Among these techniques, segmentation of medical images is a crucial step in gathering anatomical information for diagnosis or intervention planning [10].

Medical image segmentation seeks to connect a pixel with a label in a medical image without the need for human initialization. Similarly, segmentation extracts renal volume or a urinary stone from CT images and provides typical samples for nephrologists. Clinical diagnosis, radiation planning, and interventional guiding all benefit from medical image segmentation of organs. In recent years, there has been a significant increase in the development of computer-assisted technologies to aid therapists with this time-consuming activity [11]. The pixel-level semantic information helps intelligent systems to grasp spatial positions or make important judgments. Many real-world applications, including computer-aided diagnosis, benefit from this task [12,13].

Therefore, this article aims to address the automatic segmentation of kidneys and kidney stones in unenhanced CT images using five state-of-the-art deep learning algorithms. However, the success of segmentation algorithms depends on the availability of high-quality, well-annotated, and balanced imaging datasets provided by experts [14,15]. To address the data scarcity, we constructed a well-annotated abdominal CT dataset for segmentation of kidneys and kidney stones and made it public to the research community. To the best of our knowledge, we are the first to open-source our composed abdominal CT dataset of kidney and kidney stones for further research. Our experimental results also show that training deep semantic segmentation models with a proper strategy improves segmentation outcomes in abdominal CT images.

## 2. Related Work

There have been various segmentation tasks associated with kidneys, such as kidney segmentation [16], cyst segmentation [17], tumor segmentation [18], and cortex segmentation [19]. Manual kidney segmentation, such as region-of-interest (ROI) border tracing [20] or stereology [21] by experienced and qualified professionals, is considered the gold standard. However, because of the comparable signal intensities across the kidneys’ surrounding organs, and imaging artifacts, these manual methods are time-demanding (lasting 15–30 min) and might be skewed by investigator judgment [22]. An automated deep learning system that provides automatic segmentation would be a valuable supporting tool for clinicians [23,24]. Recently, artificial intelligence (AI) and deep learning (DL) methods such as convolutional neural networks (CNNs) have achieved promising results with the detection of abnormalities in a variety of medical imaging modalities, including X-ray, CT, positron emission tomography (PET), dermoscopy, ultrasound, and MRI [25,26,27,28,29].

Several segmentation methods related to kidneys and the renal tract were investigated by utilizing various imaging modalities. For example, an automated kidney segmentation task [29] was performed on MRI images using the Mask R-CNN [30]. The proposed work focused on validating a Mask R-CNN for the automated segmentation of kidneys. In one study, a 2D convolutional neural network was applied to an MRI modality to segment left and right kidneys in healthy and chronic kidney disease subjects [22]. Timothy et al. developed a convolutional neural network to apply an automated deep learning approach for cyst segmentation in MRI images [31]. The proposed system differentiated and analyzed renal cysts in patients. Adaptive sub-regional evolution level set modelling (ASLSM) is a method introduced for kidney tumor segmentation in ultrasound images [32]. The authors claimed that ASLSM was more accurate in kidney tumor segmentation than traditional ultrasound segmentation methods. Will et al. used MRI scans to perform automatic segmentation and volumetry analysis of the entire kidneys and their internal structures (cortex, medulla, and pelvis) [33]. The kidneys were separated into compartments using an automated technique based on thresholding and shape detection. Their findings showed that precise automatic segmentation of the kidneys and their interior components is possible. Similarly, several published studies used ultrasound imaging to segment kidneys [32,34,35,36,37,38], and a few detected kidney stones [39].

Apart from MRI and ultrasound images, another choice for segmentation is the CT imaging modality. CT scans, which may be performed with or without contrast, can provide more information about the renal tract. Renal CT images are used for various segmentation tasks, such as renal segmentation [40,41], urinary bladder segmentation [42], renal tumor segmentation [43], renal vessels segmentation [44], and renal stone segmentation [4]. In one study, active shape models (ASMs) with non-rigid image registration were used to segment kidneys [40]. Khalifa et al. proposed a new 3D segmentation approach to segment kidneys using CT images [41]. To distinguish between the inside and outside of the urinary bladder, a deep-learning convolutional neural network (DL-CNN) segmentation was introduced [42]. Zhao et al. presented a multi-scale supervised 3D U-Net (MSS U-Net) to segment kidneys and kidney tumors from CT images [43]. The architecture produced results superior to state-of-the-art techniques, with the Dice coefficients of kidney and tumor up to 0.969 and 0.805, respectively. Taha et al. developed a kidney vessels segmentation network with a training scheme that handled unbalanced data, reduced false positives, and enabled high-resolution segmentation with a limited memory usage [44].

A closely related work focused on the accuracy of a cascading CNN for urinary stone detection on unenhanced CT images [4]. It evaluated the performance of pre-trained models enriched with labeled CT images. The proposed model developed two CNNs: CNN1 for urinary tract detection and CNN2 for urinary stone detection. However, they borrowed the pre-trained weights from ImageNet and fine-tuned them on GrayNet (an in-house–built dataset). The GrayNet pre-trained model was then used for weight initialization of the CNN models for urinary tract identification and stone detection. In one study, the authors applied a 3D U-Net model to segment the kidneys, followed by gradient-based anisotropic denoising, thresholding, and region growing [45]. A 13-layer convolutional neural network classifier was then applied to distinguish kidney stones from false positive regions. For patient-level classification, the system achieved sensitivity of 0.88 and specificity of 0.91 on an external validation set. In contrast, our work proposes a two-stage replaceable scheme (dependent segmentation), uses a combination of state-of-the-art algorithms with a new training strategy, and improves segmentation performance.

## 3. Materials and Methods

### 3.1. Data Acquisition and Annotations

#### 3.1.1. Data Acquisition

Around 500 unenhanced abdominal CT scans from 2018 to 2020 were collected from the First Affiliated Hospital of Guangzhou Medical University. After data desensitization and data cleaning, 260 CT scans were selected for data annotations. A total of 119 scans had a thickness of 2.50 mm, 101 scans had a thickness of 1.25 mm, 31 scans had a thickness of 2.00 mm, 7 scans had a thickness of 0.62 mm, and 2 scans were 0.42 mm thick. The range of XY space was [0.371, 0.977], and the XY shape was 512 × 512. There were 209 scans with stones and 51 scans without stones. This work is a retrospective study and has been granted a waiver by the Ethics Committee of the First Affiliated Hospital of Guangzhou Medical University.

#### 3.1.2. Data Processing

The collected CT data was in DICOM format which contained patients’ personal information. To protect patient privacy and simplify labeling, it is essential to clean the data. For this purpose, SeriesID was used in the DICOM header as a reference to remove the duplicate data. Next, a Python script was implemented to delete patient personal information and saved the DICOM CT data in NIfTI format which is a standard format for storing medical images. Furthermore, due to certain constraints, the labeled cases were renamed and assigned unique identification numbers, for example, “wd0001”, “wd0011”, “wd0111”, as in the work of Heller et al. [46].

#### 3.1.3. Data Annotations

**Figure 1:**
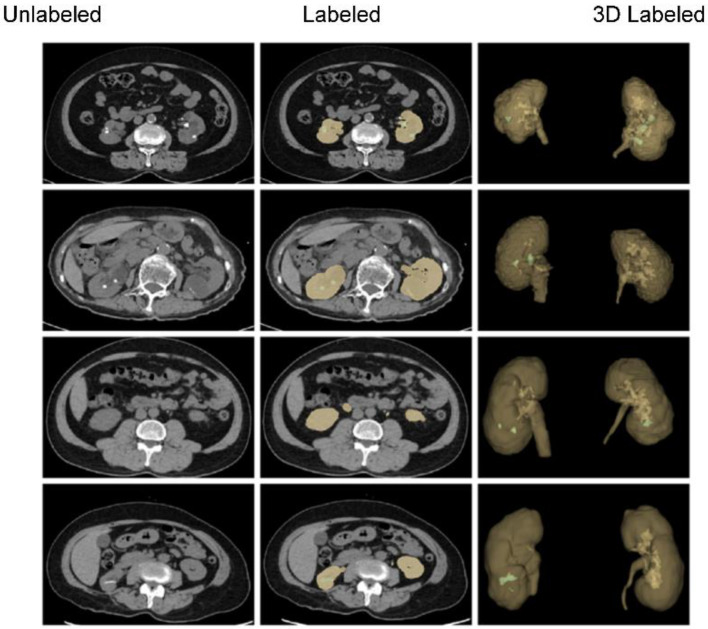
A few of the samples in both 2D and 3D from our annotated dataset of unenhanced abdominal CT images (kidney: yellow; kidney stone: green).

The clean NIfTI images of abdominal CT scans were annotated based on clinical requirements and image segmentation needs. Two labels (“kidney” and “kidney stone”) were considered for masking and labeling. The 3D Slicer [47] tool was used to mask the CT scans. The data annotations process comprised three stages. In the first stage, 30 students from biomedical engineering and computer science disciplines were trained to add the annotations. In the second stage, experienced instructors reviewed the annotations in detail, indicated potential errors, and modified them immediately. Finally, ten professional urological radiologists inspected the annotated data and suggested further modifications. The annotated data were repeatedly modified until the radiologists approved. By the end, 260 masked unenhanced CT scans were obtained in NIfTI format. Figure 1 shows abdominal CT images (original/unlabeled, labeled, and 3D labeled) from four different patients in the annotated dataset. Because the location, size and shape of kidneys and kidney stones vary considerable across patients, the segmentation of kidneys and kidney stones in CT images is challenging.

#### 3.1.4. Data Split and Preparation

**Figure 2:**
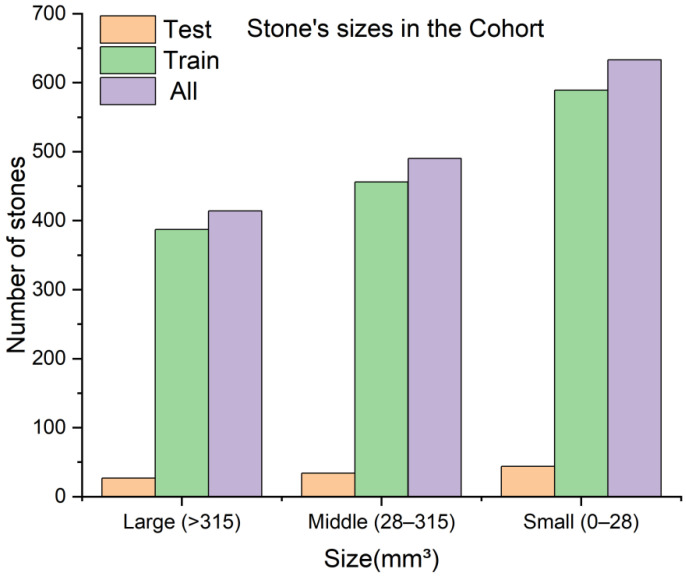
The number of kidney stones and their sizes in the training and test sets.

The annotated data was divided into training and test sets in a 7:3 ratio with 5-fold cross-validation. After data splitting, the number and volumes of all kidney stones in both the training and test sets were calculated. At the same time, based on the clinical experience, the stones were classified into small (0–6 mm), medium (6–20 mm), and large (>20 mm) sizes. Their corresponding volumes range from 0–28 mm^3^ for small, 28–315 mm^3^ for medium, and >315 mm^3^ for large stones. A detailed statistical analysis of the stones and their sizes is illustrated in Figure 2.

#### 3.1.5. Data Augmentation

To increase the model’s robustness and generalization, data augmentation was applied to the input data. However, to balance positive and negative samples, the data augmentation was only applied to labeled cubes. For instance, the first stage applied three types of data augmentation procedures to those cubes which were clipped and labeled. These procedures obtained 10,874 cubes of size 196×96×96 as input for the first segmentation network. Next, for the second stage, 3492 labeled cubes of size 160×160×64 were generated by applying the same three data augmentation techniques. These techniques include random affine transformation, horizontal inversion, and horizontal inversion + random affine transformation. The angle of the random affine transformation was 0 ± 10°, and the probability of horizontal inversion was 1.

### 3.2. Proposed Two-Stage Training Scheme

**Figure 3:**
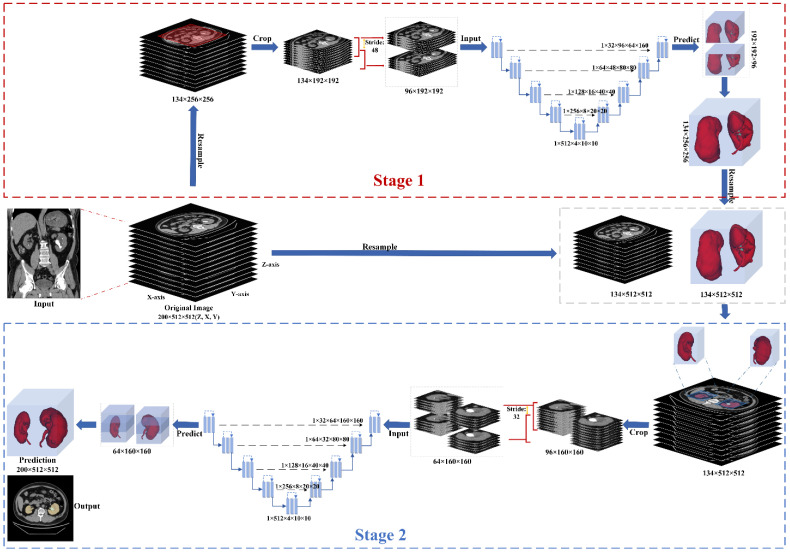
The proposed training scheme consists of two segmenting stages. In the first stage, the original image has been resampled and cropped into a single region covering both the kidneys. This cropped region is further clipped into cubes for the first segmentation network. The second stage receives the input based on the first stage and original image, which the second segmentation network could use for the fine kidney segmentation and kidney stone detection.

The intended segmentation system consists of a two-stage segmentation scheme (dependent segmentation) based on replaceable segmentation networks such as 3D U-Net [48], Res U-Net [49], SegNet [50], DeepLabV3+ [51], and UNETR [52]. Various pre- and post-processing procedures were used for the proposed training scheme, including resampling the original CT scans at a low resolution and then cropping the center regions of those CT images. The cropped CT region was then used as an input to the first segmentation network for coarse kidney segmentation. Note that the obtained segmented coarse region (containing both the coarse kidneys) was resampled to its original resolution. Next, a 3D cube containing each kidney was cropped from the original image according to the first stage’s prediction. Each cropped cube then became the input to the second segmentation network for fine kidney segmentation and kidney stone detection. Lastly, the predicted masks of the kidney and kidney stone were overlaid in the final output image. Our proposed two-stage training scheme is illustrated in Figure 3.

#### 3.2.1. Preprocessing

Due to the image size, voxel spacing, and class ratio variation, setting up three-dimensional (3D) biomedical imaging data for task-specific design and configuration of a method requires high levels of expertise and experience [53]. Thus, as an initial preprocessing step, the CT intensities in Hounsfield units (HU) were adjusted to a range of −135 to 215 or training and validation sets. The motivation behind this step was to alter the appearances of the images and highlight particular structures [8] inside the images. In addition, we analyzed the spacing distribution of the CT images and found different *z*-axis spacings between cases in the range [0.42–2.5 mm]. To equalize the *z*-axis spacing between each case, the *z*-axis spacing was adjusted to 1.25 mm.

Additionally, min–max normalization was employed to normalize the HU intensities. As the proposed training scheme consisted of two stages, in the first stage to segment the kidney, the x- and y-axes of the input data were resized to size (256, 256). Likewise, in the second stage to segment the kidney and kidney stone, the respective regions of the preprocessed images (512, 512) were cropped according to the predicted labels (masks) which were obtained from the first stage and provided as input to the second segmentation network. We used a similar approach in our previous works [54,55] which was quite successful for kidney, tumor, and cyst segmentation.

#### 3.2.2. Coarse Kidney Segmentation (Stage 1)

**Figure 4:**
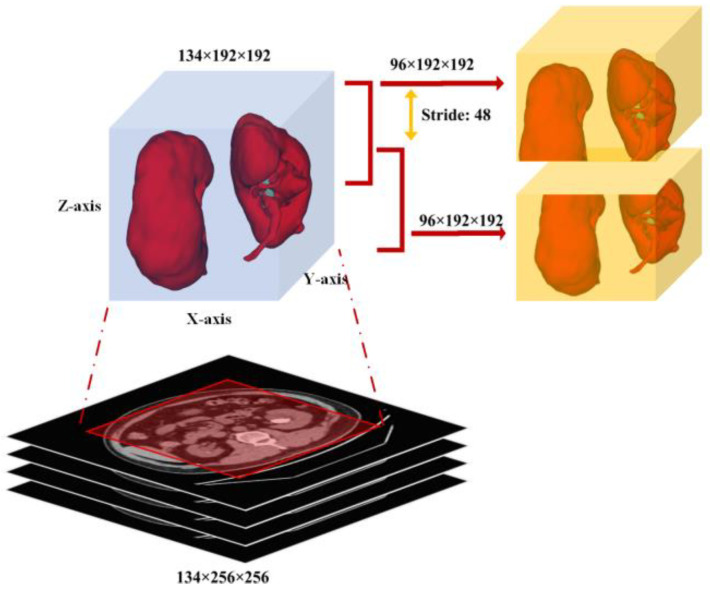
Each cropped cube with shape (1, 96, 192, 192) as input to the first segmentation network for coarse kidney segmentation.

This stage is designed to extract total kidney volumes because the dataset contains more than one class (i.e., “kidney” and “kidney stone”). However, it is not easy to directly detect or segment the kidney and kidney stone. Therefore, the masks of individual kidneys and kidney stones were changed into a single class (e.g., “kidney”), and the entire region was cropped using the center crop on the x- and y-axes with the shape (1, z, 192, 192), with the *z*-axis remaining unchanged. Next, the subsequent 3D cubes were clipped to a length of 96, stride 48, and shape (1, 96, 192, 192). For this purpose, the regionprops function was used to analyze all the input data’s kidney regions and various shape sizes were tested. The shape size (1, 96, 192, 192) was a perfect range to cover both kidney regions entirely, as shown in Figure 4. Lastly, a replaceable segmentation architecture was used with the proposed training scheme for coarse kidney segmentation, which predicted the total kidney volumes.

#### 3.2.3. Fine Kidney and Kidney Stone Segmentation (Stage 2)

**Figure 5:**
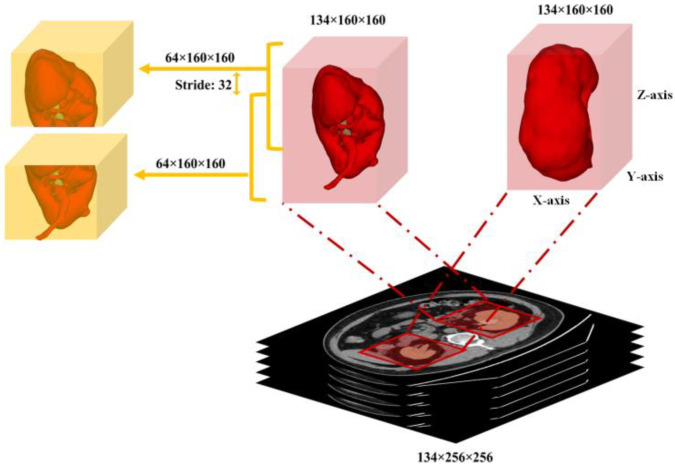
Each cropped cube with shape (1, 64, 160, 160) as input to the second segmentation network for fine kidney and stone segmentation.

For the fine kidney and stone segmentation, the regions of interest of the input preprocessed images (512, 512) were cropped according to the predicted labels (masks) from the first segmentation stage. Again, the center crop was used for cropping each particular region on the *x* and *y*-axis with the shape (1, z, 160, 160). In the subsequent step, the respective 3D cubes were clipped to a length of 64, stride 32, and shape (1, 64, 160, 160) and provided as input to the second segmentation network. Note that the same regionprops function was utilized to choose the cropping range. The proposed kidney cropping and 3D cubes clipping procedure is shown in Figure 5.

### 3.3. Adopted Segmentation Networks

For the intended training scheme, five state-of-the-art segmentation algorithms, 3D U-Net [48], Res U-Net [49], SegNet [50], DeepLabV3+ [51], and UNETR [52] were considered for training. Initially, these algorithms were trained along with the proposed training scheme. Later on, these models were trained independently based on the provided settings. Before the training procedure, a short overview of each segmentation network concerning their network architecture as follows.

The 3D U-Net is an end-to-end learning network that segments a 3D volume from sparsely annotated volumetric images employing semi- and fully automated setups. The proposed network treats 3D volumes as input and processes them with the corresponding 3D operations, particularly 3D convolutions, 3D max pooling, and 3D up-convolutional layers. The 3D U-Net avoids bottlenecks in the network architecture and uses batch normalization for faster convergence. The 2D U-Net [54] inspired the proposed architecture, replacing all 2D operations with their 3D counterparts. The proposed network has proven the ability of 3D segmentation for the analysis of medical images.

Res U-Net proposes to extract a road from images. The network was built with residual units and gained adaptability from the U-Net architecture with twofold benefits. For instance, taking advantage of the residual units, the training of deep architectures is simplified. Secondly, the presence of rich skip connections within the network ensures information propagation, which is helpful to design such networks with fewer parameters and without sacrificing the model’s performance.

SegNet is a fully convolutional neural network architecture for semantic pixel-wise segmentation. SegNet’s core trainable segmentation network consists of an encoder and decoder network followed by a pixel-wise classification layer. For the pixel-wise classification, the decoder network maps the low-resolution encoder feature maps to full input resolution feature maps. Thus, it has been considered the main novelty of the SegNet.

DeepLabV3+ uses two types of neural networks for semantic segmentation by proposing the spatial pyramid module and the encoder–decoder structure. The spatial pyramid captures rich contextual information through pooling operations at different resolutions, and the encoder–decoder architecture gradually obtains clear object boundaries. DeepLabV3+ is based on the Xception model [56]. It applies deep separable convolution to atrous spatial pyramid pooling (ASPP) and the decoder module, which results in a faster and more effective encoder–decoder network. Note that the original DeepLabV3+ architecture is 2D.

U-Net TRansformers (UNETR) is a novel architecture that uses a transformer as an encoder to learn the sequence representations of the input volume. The proposed architecture successfully captures the global multi-scale features while adhering to the famous “U-shaped” network for the encoder and decoder modules. The transformer encoder is linked directly to a decoder through skip connections at various resolutions to compute the final semantic segmentation outputs. The proposed architecture has been validated on state-of-the-art benchmarks and has yielded an excellent semantic segmentation performance.

### 3.4. Segmentation Network Training with Proposed Training Scheme

**Figure 6:**
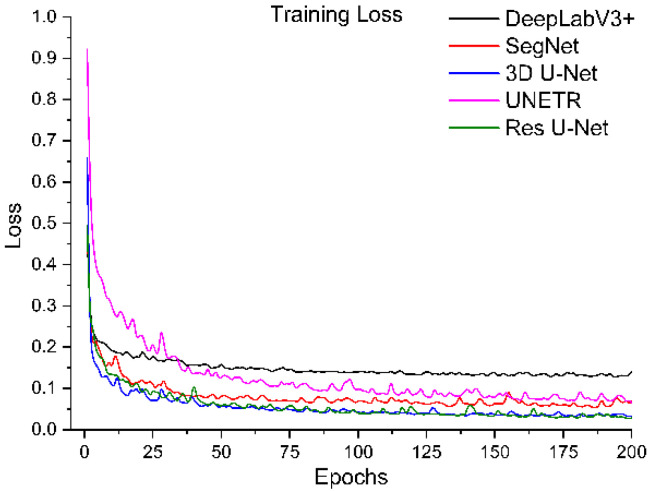
Training loss of segmentation networks trained with the proposed training scheme.

**Figure 7:**
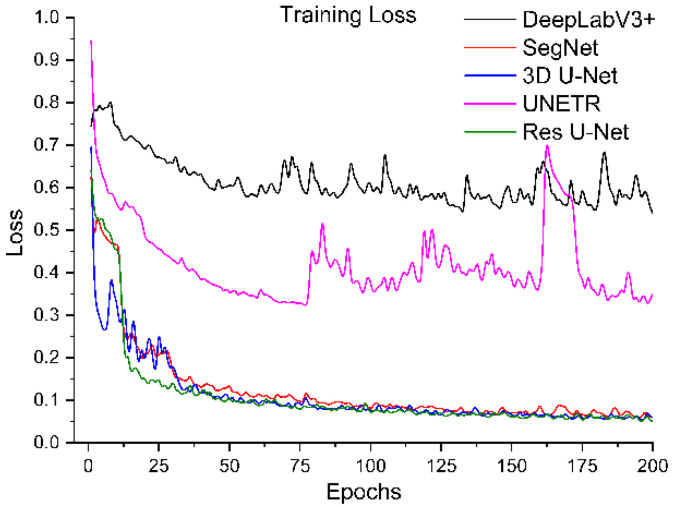
Training loss of segmentation networks trained independently.

**Figure 8:**
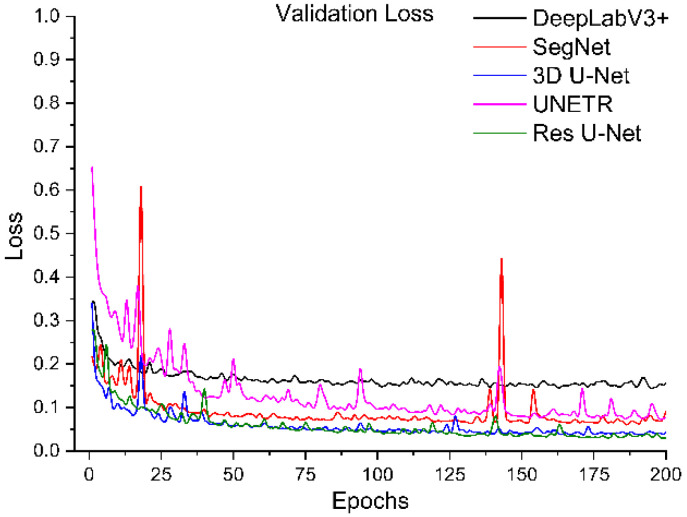
Validation loss of segmentation networks trained with the proposed training scheme.

**Figure 9:**
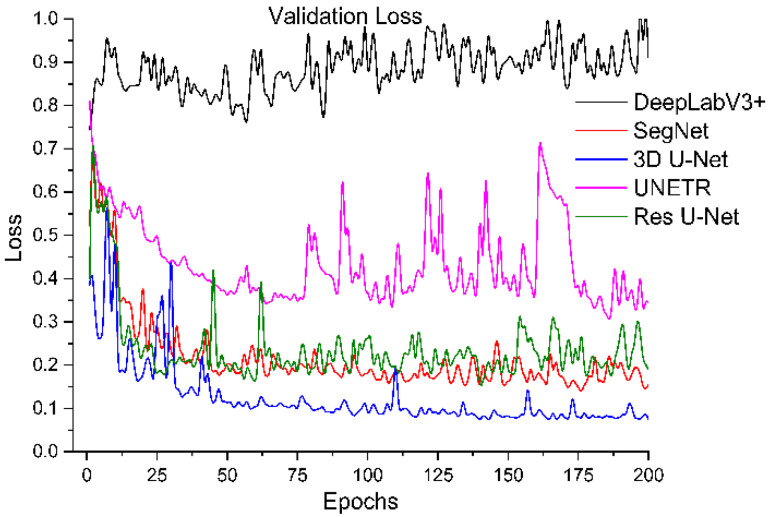
Validation loss of segmentation networks trained independently.

All segmentation networks (3D U-Net, Res U-Net, SegNet, DeepLabV3+, and UNETR) were trained for 200 epochs with AdamW [57] with batch size 8 and weight decay 0.001. The reason for using AdamW was that it exhibited better optimizing effects than conventional Adam. ReduceLROnPlateau was used to adjust the learning rate (the initial learning rate was 0.001). The Dice and cross-entropy were combined and used as a loss function. After training, the best models were selected based on validation loss. The training loss and validation loss of all models are shown in Figure 6, Figure 7, Figure 8 and Figure 9.

## 4. Results and Discussion

To train the segmentation networks independently (one-step direct segmentation) and dependently (two-step coarse-to-fine segmentation), we utilized 260 CT scans of our annotated dataset. Additionally, we applied data augmentation to produce more training samples. As mentioned above, we used 5-fold cross-validation with a batch size of 8 due to the high memory requirements. Our implementation was in Python, using the PyTorch framework (version 1.9.0) to train the model and inference the predictions. All training experiments were conducted on a GPU server with specifications of 64 GB RAM, a 2.15 GHz AMD EPYC 7742 CPU, and a Nvidia Tesla A100 GPU with 40 GB VRAM. To verify and compare the effectiveness of all trained deep learned models, the performance analysis was provided by various means. Initially, the quantitative values of the predictive models were measured in terms of Dice, specificity, sensitivity, and accuracy. These metrics are all based on True Positive (TP), False Positive (FP), True Negative (TN) and False Negative (FN) with definitions as follows:

$$\mathrm{Dice}=\frac{2\mathrm{TP}}{2\mathrm{TP}+\mathrm{FP}+\mathrm{FN}}$$

$$\mathrm{Specificity}=\frac{\mathrm{TN}}{\mathrm{TN}+\mathrm{FP}}$$

$$\mathrm{Sensitivity}=\frac{\mathrm{TP}}{\mathrm{TP}+\mathrm{FN}}$$

$$\mathrm{Accuracy}=\frac{\mathrm{TP}+\mathrm{TN}}{\mathrm{TP}+\mathrm{TN}+\mathrm{FP}+\mathrm{FN}}$$

To systematically compare the performance of these five segmentation networks with our proposed training scheme, we trained them ten times and calculated the evaluation metrics mentioned above. The mean and standard deviation of these metrics for ten evaluations of each model are shown in Table 1. It shows that Res U-Net performs better than U-Net, followed by the other trained models. The DeepLabV3+ and UNETR predictions are not satisfactory, referring particularly to the kidney stone Dices. Assessing the performances of the models trained independently, Table 2 has lower percentages than the performances of the trained models with our proposed training scheme. Again, Res U-Net surpassed all the other models in terms of evaluation metrics when trained independently. Likewise, the quantitative performances of other independently trained models follow the same performance order as training by the proposed training scheme. Further, each trained model was validated by their training and validation loss. Figure 6 and Figure 7 compare the training losses of all segmentation networks trained dependently and independently. The validation loss comparison of each trained network appears in Figure 8 and Figure 9, verifying the quantitative analyses provided in Table 1 and Table 2.

**Figure 10:**
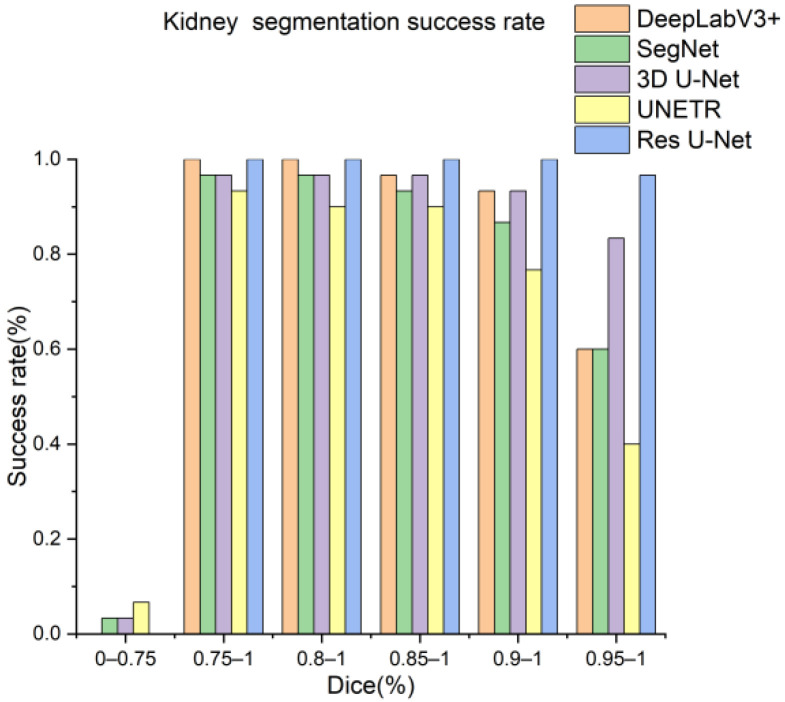
Kidney segmentation success rate of segmentation networks with proposed training scheme.

**Figure 11:**
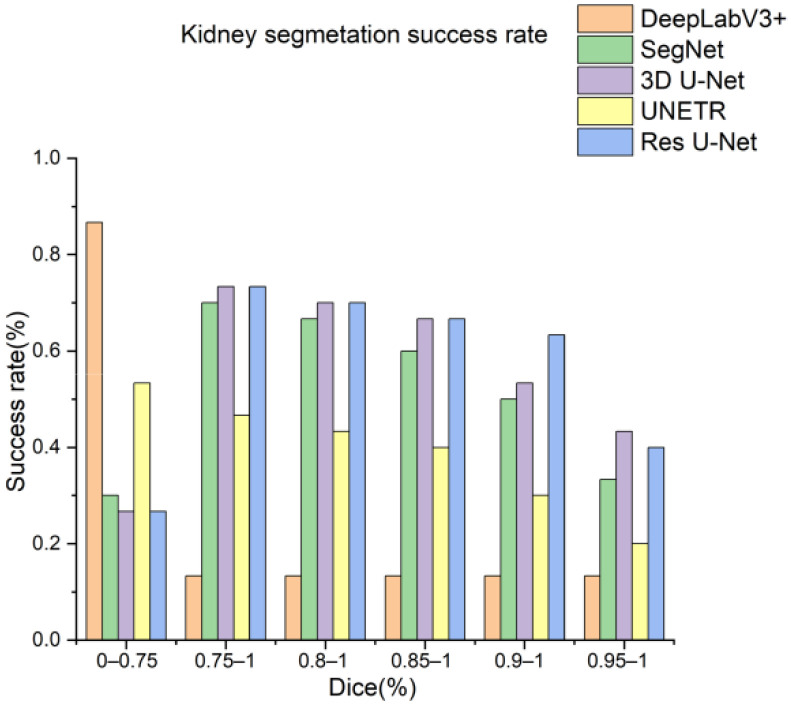
Kidney segmentation success rate of segmentation networks trained independently.

**Figure 12:**
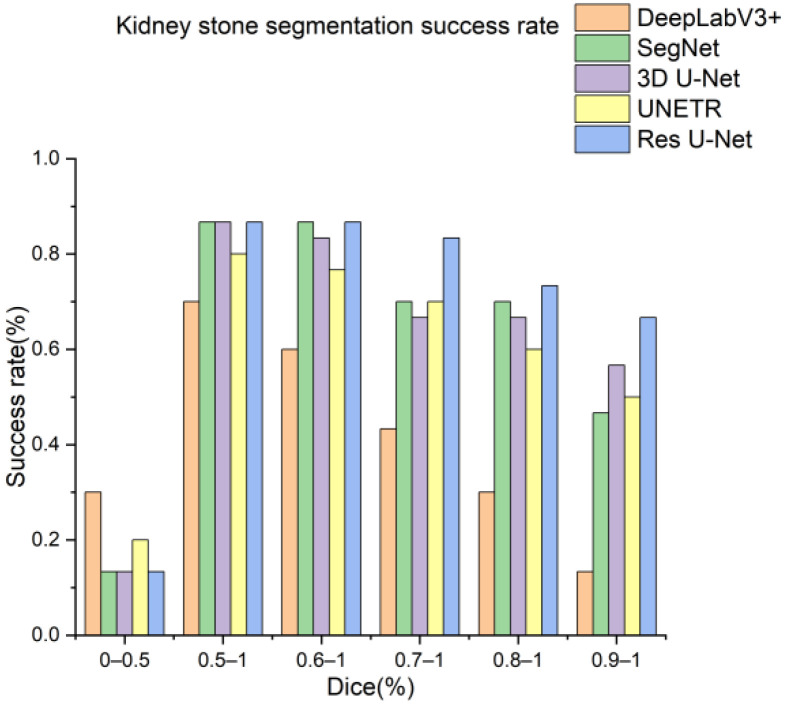
Kidney stones segmentation success rate of segmentation networks trained with the proposed training scheme.

**Figure 13:**
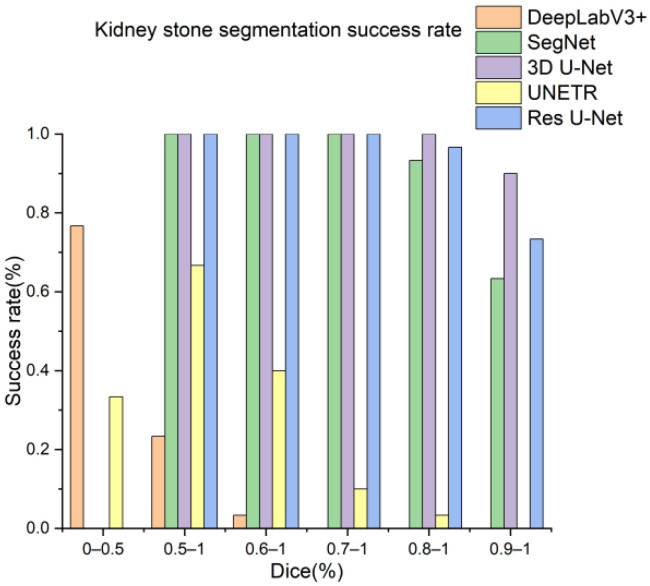
Kidney stones segmentation predictions success rate of segmentation networks trained independently.

**Figure 14:**
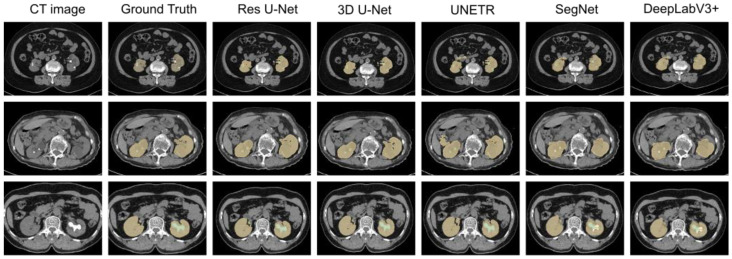
The 2D visual analysis of segmented kidneys and kidney stones predicted by the models trained dependently.

**Figure 15:**
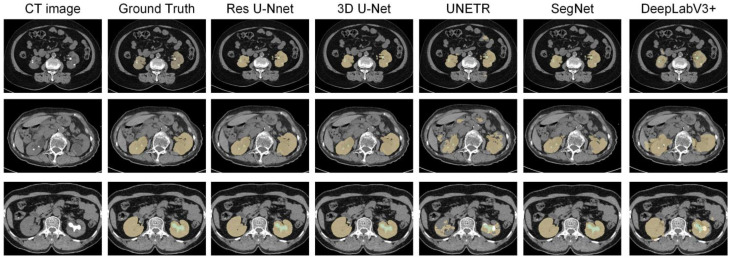
The 2D visual analysis of segmented kidneys and kidney stones predicted by the models trained independently.

**Figure 16:**
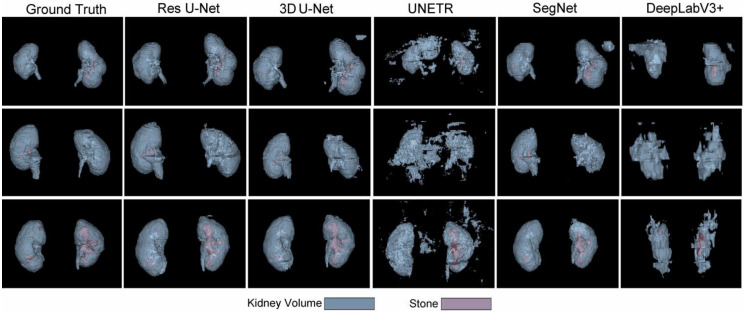
The 3D visual analysis of predictions generated by the models trained dependently.

**Figure 17:**
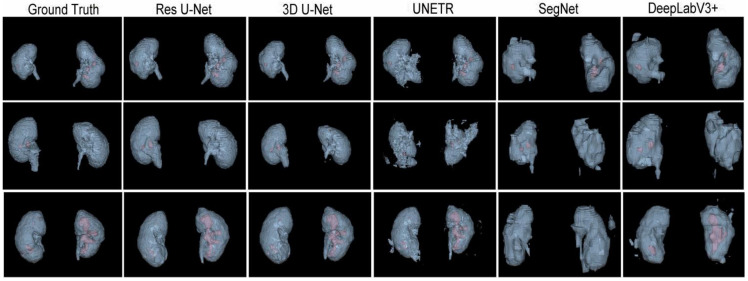
The 3D visual analysis of predictions generated by the models trained independently.

Next, the trained models were assessed using the success rate of the test set. The success rate is the proportion of successful efforts to accomplish an operation or task. For this purpose, the generated predictions were categorized into kidney and kidney stone segmentation predictions. The respective success rates of all independent and dependent trained models are provided in Figure 10, Figure 11, Figure 12 and Figure 13. Lastly, the final imaging outputs were analyzed and compared. Figure 14 and Figure 15 show the 2D segmented predictions, and Figure 16 and Figure 17 show the 3D visualizations of segmented kidneys and kidney stones.

A comparison of Figure 14 and Figure 15 shows that the models trained with the proposed training procedure gave better segmented predictions. The Res U-Net showed the highest performance, followed by other trained networks using test data. The 3D U-Net was a close competitor to Res U-Net. Otherwise, DeepLabV3+, UNETR, and SegNet missed some of the target regions belonging to kidneys and kidney stones. Both Figure 14 and Figure 15 can be expanded to view the details more clearly. Figure 16 and Figure 17 illustrate the 3D visual quality evaluation of kidney and stone segments generated by the segmentation networks trained dependently and independently. These visual evaluations illustrate that the segmented images generated by the segmentation networks applied with the proposed training method are more prominent than the 3D segments extracted by the independently trained models. Similarly, Figure 10 shows that the most effective segmentation algorithms were, again, Res U-Net and 3D U-Net, followed by SegNet, UNETR, and DeepLabV3+. The 3D segments produced by Res U-Net and 3D U-Net are quite close to respective ground truth, while those produced by DeepLabV3+, SegNet, and UNETR yielded outputs with missing or inaccurate predictions. Referring to Figure 11, which shows their visualized 3D outputs, the performances of DeepLabV3+, SegNet, and UNETR are, again, considerably defective compared to Res U-Net and 3D U-Net.

In addition, we show the Dice results by kidney stone size to establish whether the size of the stone impacts the segmentation performance in Table 3. We prove that the results are better in segmentation for larger kidney stone. The experimental results prove that the proposed two-stage training scheme performs much better than the segmentation networks which are trained independently. The results also show that kidney stone detection is harder for the segmentation networks due to varying sizes, diversity in appearance, small shapes, and many negative samples in an abdominal CT scan. In the same way, entire kidney detection and segmentation becomes challenging due to their deformable shapes from slice to slice. Therefore, the two-stage segmentation networks strategy is ideal where the entire kidney region has been extracted initially as an input for the first segmentation network. The second stage includes cropping the kidneys separately and then providing them as an input to the second segmentation network. The network ignores many irrelevant features in abdominal CTs and thus simplifies kidney stone segmentation.

A notable trade-off regarding the proposed two-stage training procedure is the heavy requirement of computing resources. In addition, the second stage relies heavily on the segmented outputs produced by the first stage’s segmentation network. For example, a false positive can affect the entire kidney region’s cropping ability, leading to incorrect predictions. However, the probability of such a scenario is quite rare as kidney extraction is easier than kidney stone detection. Thus, to avoid such challenges, we intend that our future prediction model will utilize a single segmentation network without scarifying the finely segmented predictions.

## 5. Conclusions

Contemporary benchmark challenges are increasingly dominated by machine learning and deep learning techniques with convolutional neural networks, such as the Kidney and Kidney Tumor Segmentation (KiTS19) challenge, which appeared in the MICCAI 2019. Recent machine automation trends have also extensively inspired and aided biomedical engineering and clinical practices. There has been less attention paid to automatic kidney segmentation and kidney stone detection using CT images. Consequently, this work focused on performing kidney and kidney stone detection tasks and presented a replaceable training procedure for 3D semantic segmentation algorithms. The segmentation algorithms trained with the proposed training strategy performed considerably well in terms of Dice, specificity, sensitivity, and accuracy. Moreover, this work contributes to an open-access well-annotated abdominal CT dataset of kidneys and kidney stones for further research. It would be among the primary works which address kidney segmentation and kidney stone detection challenges using the CT images. We believe that this work will also have a real-time impact, such as preoperative planning for percutaneous nephrolithotomy (PCNL). Thus, such techniques could be extended for PCNL surgical intervention. However, to fully leverage such techniques for PCNL surgical intervention, there is a need to explore the kidney’s inner structure in more detail, such as by taking advantage of the enhanced CT images and then training the intelligent models accordingly. Inspired by these goals, our future work aims to annotate and segment the inner kidney’s structure using enhanced CT images. This work also reveals that research on the topic is limited. To the best of our knowledge, no work provides a well-annotated open-access CT image dataset both for kidney and kidney stone segmentation and detection. In conclusion, deep semantic segmentation models are feasible and capable of accurately segmenting kidneys and detecting kidney stones on unenhanced abdominal CT scans.

## Figures and Tables

**Table 1:** The performances of the networks trained ten times with the proposed training scheme.

Network	Kidney (Mean ± Std)			Kidney Stone (Mean ± Std)		
	Dice	Specificity	Sensitivity	Dice	Specificity	Sensitivity
SegNet	95.04 ± 1.18%	99.96 ± 0.02%	95.69 ± 0.94%	75.59 ± 2.23%	99.96 ± 0.01%	73.43 ± 1.18%
DeepLabV3+	81.73 ± 2.43%	99.89 ± 0.02%	82.52 ± 2.50%	33.36 ± 3.17%	99.96 ± 0.01%	35.61 ± 3.28%
3D U-Net	96.05 ± 1.68%	99.98 ± 0.01%	96.05 ± 0.18%	80.04 ± 1.75%	99.98 ± 0.01%	80.21 ± 1.81%
UNETR	93.35 ± 1.45%	99.97 ± 0.01%	93.09 ± 0.82%	74.36 ± 0.80%	99.96 ± 0.01%	76.26 ± 2.78%
Res U-Net	**96.54 ± 1.06%**	**99.99 ± 0.01%**	**96.49 ± 0.08%**	**80.59 ± 1.28%**	**99.99 ± 0.01%**	**79.73 ± 1.90%**

**Table 2:** The performances of the networks trained independently.

Network	Kidney Stone Dice	Kidney Dice	Specificity	Sensitivity	Accuracy
SegNet	75.42%	95.50%	99.96%	97.50%	99.94%
DeepLabV3+	41.09%	65.56%	99.75%	70.91%	99.59%
3D U-Net	77.63%	96.70%	99.97%	97.20%	99.96%
UNETR	61.92%	77.14%	99.82%	82.02%	99.72%
Res U-Net	**79.83%**	**95.81%**	**99.97%**	**96.61%**	**99.95%**

**Table 3:** The performances of different networks by kidney stone size.

Network	Kidney Stone Dice (Mean ± Std)		
	Small	Middle	Large
SegNet	34.38 ± 1.67	74.76 ± 2.86	80.86 ± 4.51
DeepLabV3+	\	5.61 ± 2.48	47.42 ± 2.70
3D U-Net	58.03 ± 1.42	81.56 ± 1.99	82.86 ± 3.52
UNETR	52.49 ± 2.13	76.54 ± 2.20	76.38 ± 1.85
Res U-Net	**60.11 ± 0.84**	**76.08 ± 3.46**	**83.39 ± 2.33**

## Data Availability

To make available our annotated dataset to research community for further research, the dataset has been uploaded into the Zenodo repository at https://zenodo.org/record/6042410 (accessed on 2 June 2022). Interested users can directly download them upon request.
